# Gut microbiota is causally associated with poststroke cognitive impairment through lipopolysaccharide and butyrate

**DOI:** 10.1186/s12974-022-02435-9

**Published:** 2022-04-04

**Authors:** Huidi Wang, Mingsi Zhang, Jie Li, Jianhai Liang, Mengjia Yang, Genghong Xia, Yueran Ren, Hongwei Zhou, Qiheng Wu, Yan He, Jia Yin

**Affiliations:** 1 Department of Neurology, Nanfang Hospital, Southern Medical University, Guangzhou, Guangdong China; 2 Microbiome Medicine Center, Department of Laboratory Medicine, Zhujiang Hospital, Southern Medical University, Guangzhou, Guangdong China

**Keywords:** Post-stroke cognitive impairment, Lipopolysaccharide, Fecal microbiota transplantation, Hippocampal apoptosis, β-Amyloid

## Abstract

**Background:**

Poststroke cognitive impairment (PSCI) is prevalent in stroke patients. The etiology of PSCI remains largely unknown. We previously found that stroke induces gut microbiota dysbiosis which affects brain injury. Hereby, we aimed to investigate whether the gut microbiota contributes to the pathogenesis of PSCI.

**Methods:**

83 stroke patients were recruited and their cognitive function were measured by Montreal Cognitive Assessment (MoCA) scores 3 months after stroke onset. The peripheral inflammatory factor levels and gut microbiota compositions of the patients were analyzed. Fecal microbiota transplantation from patients to stroke mice was performed to examine the causal relationship between the gut microbiota and PSCI. The cognitive function of mice was evaluated by Morris water maze test.

**Results:**

34 and 49 stroke patients were classified as PSCI and non-PSCI, respectively. Compared with non-PSCI patients, PSCI patients showed significantly higher levels of gut *Enterobacteriaceae*, lipopolysaccharide (LPS) and peripheral inflammation markers. Consistently, stroke mice that received microbiota from PSCI patients (PSCI mice) presented a higher level of *Enterobacteriaceae*, intestinal Toll-like receptor-4 (TLR4) expression, circulating LPS, LPS-binding protein (LBP) and inflammatory cytokines, and a lower level of fecal butyrate, severer intestine destruction and cognitive impairment than mice that received microbiota from nPSCI patients (nPSCI mice). In addition, we observed exacerbations in blood–brain barrier (BBB) integrity, microglial activation, neuronal apoptosis in the CA1 region of the hippocampus, and Aβ deposition in the thalamus of PSCI mice in comparison with nPSCI mice. Intraperitoneal injection of LPS after stroke caused similar pathology to those seen in PSCI mice. Supplementation with sodium butyrate (NaB) via drinking water rescued these detrimental changes in PSCI mice.

**Conclusions:**

Our data indicate a cause–effect relationship between gut microbiota and PSCI for the first time, which is likely mediated by inflammation-regulating metabolites including LPS and butyrate.

**Supplementary Information:**

The online version contains supplementary material available at 10.1186/s12974-022-02435-9.

## Introduction

Stroke remains a major cause of death and disability globally [1]. Poststroke cognitive impairment (PSCI) is one of the most common complications of stroke and is estimated to affect one-third of stroke patients [2]. Despite its high prevalence, current treatments for PSCI are very limited [3]. Moreover, the mechanisms underlying the occurrence of PSCI remain poorly explored.

In recent years, it was found that the levels of inflammatory factors such as C reactive protein (CRP), interleukin (IL)-6, IL-1β, and IL-10 are increased in the plasma of patients with PSCI [4–6]. Since the hippocampus is selectively vulnerable to inflammation and is considered a critical brain region for learning and memory [7], it can be speculated that damage to this structure after stroke may contribute to the development of PSCI. However, how excessive peripheral inflammation develops in individuals with PSCI is not clear.

The gut microbiota is known to extensively orchestrate various aspects of human physiology, from extraction of energy from nutrients and vitamin synthesis to modulation and maintenance of nervous, metabolic and immune system stability [8–10]. In the context of stroke, brain injury can induce significant changes in the composition of the gut microbiota [11], which affects other organs [12]. Moreover, gut microbiota dysbiosis induces proinflammatory T cell polarization in the intestine, and these intestinal lymphocytes can migrate into the brain to affect stroke outcomes [13, 14]. Our previous work demonstrated that stroke triggers gut microbiota dysbiosis, hallmarked by *Enterobacteriaceae* expansion, which exacerbates brain infarction [15]. The *Enterobacteriaceae* family represents the facultative anaerobic microbes that can respire oxygen emanating from an inflamed epithelium for their growth [16]. In addition, *Enterobacteriaceae* can utilize the nitrate and electron acceptors that are generated during epithelial inflammation for anaerobic respiration, rendering them competitive in intestinal ecology [17]. Butyrate activates peroxisome proliferator-activated receptor γ (PPAR-γ) signaling and drives β-oxidation of epithelial cells, leading to decreased level of oxygen with gut lumen, which limits a dysbiotic expansion of *Enterobacteriaceae* [18]. Recently, several studies have reported that gut microbiota composition is altered in PSCI patients [19–21]. All these findings collectively suggest a potential role of gut microbiota (most likely through its inflammation-regulating metabolites) in the development of PSCI.

In the present study, we investigated the gut microbiota and inflammatory status of PSCI patients and identified that gut microbiota is associated with inflammation. To verify the causal role of the gut microbiota in the pathogenesis of PSCI, we transferred gut microbiota from PSCI patients into stroke mice by fecal microbiota transplantation (FMT) and evaluated the cognitive performance of the recipient mice and studied the pathology of the intestine and the brain to investigate the underlying mechanism.

## Methods

### Humans

The subjects were recruited from the Department of Neurology of Nanfang Hospital of Southern Medical University (Guangzhou, China) from September 2018 to December 2020. The inclusion criteria were as follows: (i) an age greater than 18 years (ii) a diagnosis within 4 days of stroke onset according to established guidelines [22], and (iii) a National Institutes of Health Stroke Scale (NIHSS) score ≤ 8. The exclusion criteria were as follows: (i) patients who presented significant neurological deficits such as drowsiness, aphasia, or limb weakness and were, therefore, unable to complete the cognitive function test (ii) patients with a history of seizures and obvious cognitive impairment (AD8 ≥ 2) before stroke [23], mental disorders or significant emotional problems; (iii) patients with infectious diseases, such as pneumonia or urinary system infection; (iv) patients administered antibiotics or probiotics within 1 month before admission or during follow-up; and (v) patients for whom stool samples could not be obtained within 4 days of admission or at the 3-month follow-up. The serum samples were isolated by centrifugation at 3000 rpm for 10 min and stored at − 80 °C until testing. All participants provided written informed consent in accordance with the Declaration of Helsinki. This study was approved by the Ethics Committee of Nanfang Hospital, Southern Medical University (NFEC-2020-169) and registered at http://clinicaltrials.gov (NCT04688138).

### Cognitive assessment

Cognitive impairment was assessed by the Montreal Cognitive Assessment (MoCA) and the Mini-Mental State Examination (MMSE) 3 months after stroke onset. MMSE and MoCA scores range from 0 to 30. The MoCA is currently the most widely recognized tool for assessing cognitive function, including visuospatial/executive function, naming, attention, abstraction, language, delayed recall, and orientation [24]. Patients with a MoCA score < 22 were considered to have PSCI [25]; higher scores indicate better cognitive performance.

### Mice

The experiments were approved by the Ethics Committee for Animal Care and Research of Zhujiang Hospital of Southern Medical University (Guangdong, China) and were performed according to the ARRIVE guidelines. As female hormone such as estrogen and follicle-stimulating hormone can significantly affect cognition [26, 27], to exclude possible influence of female hormone, male mice were used in this study. Adult male C57BL/6J mice (8–10 weeks, 22–25 g) were purchased from Guangdong Medical Laboratory Animal Center (Guangzhou, China). Upon arrival, mice were randomly divided into three groups in FMT experiment and were randomly divided into two groups in intraperitoneal injection experiment. Mice that were allocated to the same group were raised in the same cage. All animals were housed under controlled temperature and humidity conditions on a 12-h:12-h light/dark cycle and were provided food and water ad libitum. After acclimatization for 1 week, fecal samples from mice were collected and stored in a − 80 °C freezer until analysis. For antibiotic treatment, broad-spectrum antibiotics (1 g ampicillin, 1 g neomycin sulfate, and 1 g metronidazole, Sigma-Aldrich, CA, USA) were dissolved in 1 L drinking water (replace every 3 days) and provided ad libitum to the mice for 14 consecutive days, after which fecal samples were collected and stored. For sodium butyrate (NaB) treatment, 11 g NaB (Aladdin) was dissolved in drinking water to a concentration of 0.1 mol/L and provided ad libitum to the mice. Supplementation with NaB by this dose and regime effectively shapes the gut microbiota of mice [28, 29]. For lipopolysaccharide (LPS) treatment, LPS serotype 0111:B4 (Sigma-Aldrich, St. Louis, MO, USA; dissolved in phosphate-buffered saline (PBS), 5 μg/mouse) was administered intraperitoneally to the mice every 3 days. For FMT, fecal samples were collected from patients at 3 months after stroke. All individual samples from the same group were pooled together. Fecal microbiota suspensions were prepared by diluting and mixing 10 g of fecal samples obtained from PSCI or non-PSCI (nPSCI) patients in 100 mL of sterile PBS and vortexed vigorously. Then the suspensions were centrifuged at 500*g* for 1 min to remove insolubilized material and the supernatants were collected and stored in a − 80 °C freezer until further use. Three days after stroke, each mouse was intragastrically administered 0.2 mL of the suspension once daily for 28 consecutive days.

### Cerebral ischemia and reperfusion model

Focal cerebral ischemia was induced by transient (30 min) middle cerebral artery occlusion (MCAO) using an intraluminal filament as previously described [28]. Surgical anesthesia was induced by intraperitoneal injection of 1.25% tribromoethanol (0.02 mL/g of body weight). Body temperature was maintained throughout the procedure with a feedback-controlled heating blanket. A filament was introduced into the external carotid artery and gently advanced into the internal carotid artery until it reached the middle cerebral artery. After 30 min of cerebral ischemia, the filament was withdrawn to establish reperfusion. Two Mice were excluded due to absence of deficits in neurological function (modified neurological severity score ≤ 1). Two mice died after surgery, one mouse was excluded due to subarachnoid hemorrhage during surgery.

### Neurological severity and cognition assessment

Neurological function was assessed by the modified neurological severity score (mNSS) [28]. The mice were tested 3 days after experimental stroke. The mNSS is a composite of motor (muscle status and abnormal movement), sensory (visual, tactile, and proprioceptive sensations), and reflex tests. Neurological severity was graded on a scale of 0 to 18 points. A higher score indicated a more severe brain injury. Cognitive functions were assessed by the Morris water maze on the next day after FMT or intraperitoneal injection. The mice were subjected to four trials per day for 5 consecutive days. In all trials, the mice were introduced into a pool (120 cm in diameter and 50 cm high) containing a 10-cm-diameter platform submerged 1 cm below the surface of the water in quadrant III. In this test, the mice searched for the hidden platform by using memory of visual cues around the pool. Each mouse was put in the water facing the pool wall and given 60 s to find the hidden platform. If a mouse failed to find the platform within a limited time (60 s), it was guided to swim to the platform and kept there for 20 s. The time taken to reach the platform (escape latency) was measured. On the 6th day of trails, a 60-s probe trial was conducted to evaluate the memory of the mice. The number of times the mice crossed the area of platform and the percentage of time staying in the target quadrant (quadrant III) were recorded with a digital video camera.

### 16S RNA sequencing and analysis

Bacterial genomic DNA was extracted using a QIAamp PowerFecal Pro DNA Kit (QIAGEN, Valencia, CA, USA) according to the manufacturer’s instructions. The barcoded primers V4F (GTGYCAGCMGCCGCGGTAA) and V4R (GGACTACNVGGGTWTCTAAT) were used to amplify the V4 variable region of the 16S rRNA gene. PCR was performed according to a previously described method [15]. All PCR amplicons were mixed and sequenced using the Illumina iSeq 100 platform. The Shannon index, phylogenetic diversity (PD) whole-tree index, and Chao1 index were determined to assess α-diversity. UniFrac distances were used to analyze the β-diversity by illustrating the phylogenetic dissimilarity among samples. A smaller UniFrac distance between two samples indicates a higher similarity. As a dimensionality reduction method, principal-coordinate analysis (PCoA) was used to describe the relationships among samples based on the distance matrix and visualize the unsupervised grouping pattern of the complex data set, i.e., the microbiome. Linear discriminant analysis effect size (LEfSe) was used to compare the discriminative data between groups.

### Extraction and quantification of short-chain fatty acid (SCFA) levels

Approximately 0.2 g of feces was homogenized in 1 mL of ultrapure containing an internal standard of 2,2-dimethylbutyric acid. The homogenate was centrifuged at 12,000 rpm for 10 min at 4 °C. Then, the supernatant was transferred to another tube and mixed with 10 mL of 50% sulfuric acid, 2 mL of analytically pure diethyl ether, and 0.5 g of sodium sulfate (Macklin, China). The mixture was vigorously vortexed for 1 min and then centrifuged at 5000 rpm for 10 min at room temperature. The ether layer was collected for gas chromatography with mass selective detection (5977B GC-MSD system; Agilent Technologies, Santa Clara, CA, USA). An HP-free fatty acid phase capillary column was used for chromatographic separation, with helium as the carrier gas. The oven temperature was increased from 90 to 180 °C at a rate of 15 °C/min. Gas chromatography spectrometry (GC–MS) data were collected and analyzed with MassHunter Workstation software (Agilent Technologies). Final concentrations were calculated based on internal standards and are presented as micromoles per gram of wet feces (μmol/g).

### Measurements of peripheral cytokine levels

The serum levels of LPS (MM-0634M1), LPS-binding protein (LBP, MM-44515M1), d lactate (DLA, MM-50958H1), IL-6 (MM-0163M1), TNF-α (MM-0132M1) and IL-1β (MM-0040M1) were determined using enzyme-linked immunosorbent assay (ELISA) kits (Meimian, Jiangsu, China) according to the manufacturer’s protocols. The erythrocyte sedimentation rate (ESR) was measured using an automatic ESR analyzer (Electa Lab, XC-40B, Forli, Italy).

### H&E staining and Nissl staining

After cognitive assessment, the mice were anesthetized and subjected to cardiac perfusion with saline and fixation with paraformaldehyde (PFA). The ileum (1 cm from the cecum) of each mouse was harvested, dissected in a length of 1 cm, fixed with 4% PFA for 24 h, and then embedded in paraffin. Next, 4-mm-thick sections were cut, dewaxed, and stained with hematoxylin and eosin using standard protocols. For Nissl staining, brain tissue was harvested carefully, fixed with 4% PFA for 24 h, and then cryoprotected with 30% sucrose for 2 days. A 4-mm-thick serial frozen coronal section was cut using a cryostat (Leica CM1950) at − 20 °C. Then, the sections were dried in air, washed twice with distilled water, and stained with 1% toluidine blue for 5 min. The sections were washed 3 times with distilled water, placed in 70% ethanol for 2 min, washed twice with 95% ethanol, and then washed with xylene for 5 min. The sections were observed with a microscope (DM2500 microscope; Leica). Crypt depth were measured from the bottom of the crypt to the crypt-villus junction and villus length were measured from the crypt-villus junction to the tip of the villus.

### Immunofluorescence staining and TUNEL staining

Coronal brain slices prepared as described above were blocked and incubated with an anti-Iba1 (1:500; Abcam, ab178846) or anti-β-amyloid (Aβ) (1:500; Abcam, ab32136) antibody overnight at 4 °C and then with Alexa Fluor 488-conjugated goat anti-rabbit IgG (1:1000, Life Technologies) for 1 h at room temperature and counterstained with 4ʹ,6-diamidino-2-phenylindole (DAPI). Fluorescence signals were visualized with a laser scanning confocal microscope (Zeiss, Oberkochen, Germany). To measure hippocampal apoptosis, brain sections were subjected to TUNEL staining (Thermo Fisher) and counterstained with DAPI following the manufacturer’s instructions.

### Western blotting

Colon and brain tissue samples were snap-frozen in cryotubes submerged in liquid nitrogen. Tissues were ground and lysed radioimmunoprecipitation assay lysis buffer (Beyotime Biotechnology, China) containing the protease inhibitor phenylmethylsulfonyl fluoride (PMSF; Beyotime Biotechnology, China) using a homogenizer and incubated on ice for 30 min. After centrifugation at 12,000 rpm for 30 min at 4 °C, the supernatants were collected. Protein concentration was measured by BCA protein assay kit (Thermo Fisher Scientific, Waltham, MA, USA), and a total of 40 μg protein was separated by 10% SDS–PAGE and subsequently electrophoretically transferred onto a polyvinylidene difluoride membrane (Millipore, Temecula, CA, USA). The membrane was blocked with 5% nonfat milk at room temperature for 1 h and then incubated with primary antibodies against ZO-1 (1:1000; Abcam, ab216880), Occludin (1:1000; Abcam, ab167161), Claudin-4 (1: 1000; Abcam, ab53156), Toll-like receptor 4 (TLR4) (1:1000; Abmart, TA7017S) and β-actin (1:1000; Abcam, ab8226) at 4 °C overnight. The membrane was washed with Tris-buffered saline containing 0.1% Tween 20 and incubated with a horseradish peroxidase-conjugated goat anti-rabbit or goat anti-mouse secondary antibody (1:10,000; Thermo Scientific). The membranes were then visualized with an enhanced chemiluminescence system (Thermo Scientific, Rockford, IL). The band intensity was assessed using Image J software. To measure the expression of each protein, the relative intensity was calculated by comparing the intensity of β-actin, the fold change of protein expression = relative intensity of each protein/mean relative intensity of nPSCI or PBS group.

### Statistical analysis

The data are expressed as the mean ± SD. Statistical significance between the two groups was assessed using Student’s *t* test or nonparametric Mann–Whitney test. One-way ANOVA followed by the least significant difference (LSD) post hoc test or nonparametric Kruskal–Wallis test was used to compare three groups. The changes in the abundance of *Enterobacteriaceae* within patients was assessed by Wilcoxon matched-pairs signed rank test. Escape latency was compared by repeated-measure ANOVA. Correlations were analyzed by Spearman’s rank correlation. Multivariate logistic regression analyses were performed, and odds ratio (OR) and 95% confidence interval (CI) were calculated. *P* < 0.05 was considered statistically significant. Statistical analysis was performed using GraphPad Prism 8 software (GraphPad Software Inc).

## Results

### PSCI patients exhibit excess peripheral inflammation and increased abundance of *Enterobacteriaceae*

**Fig. 1:**
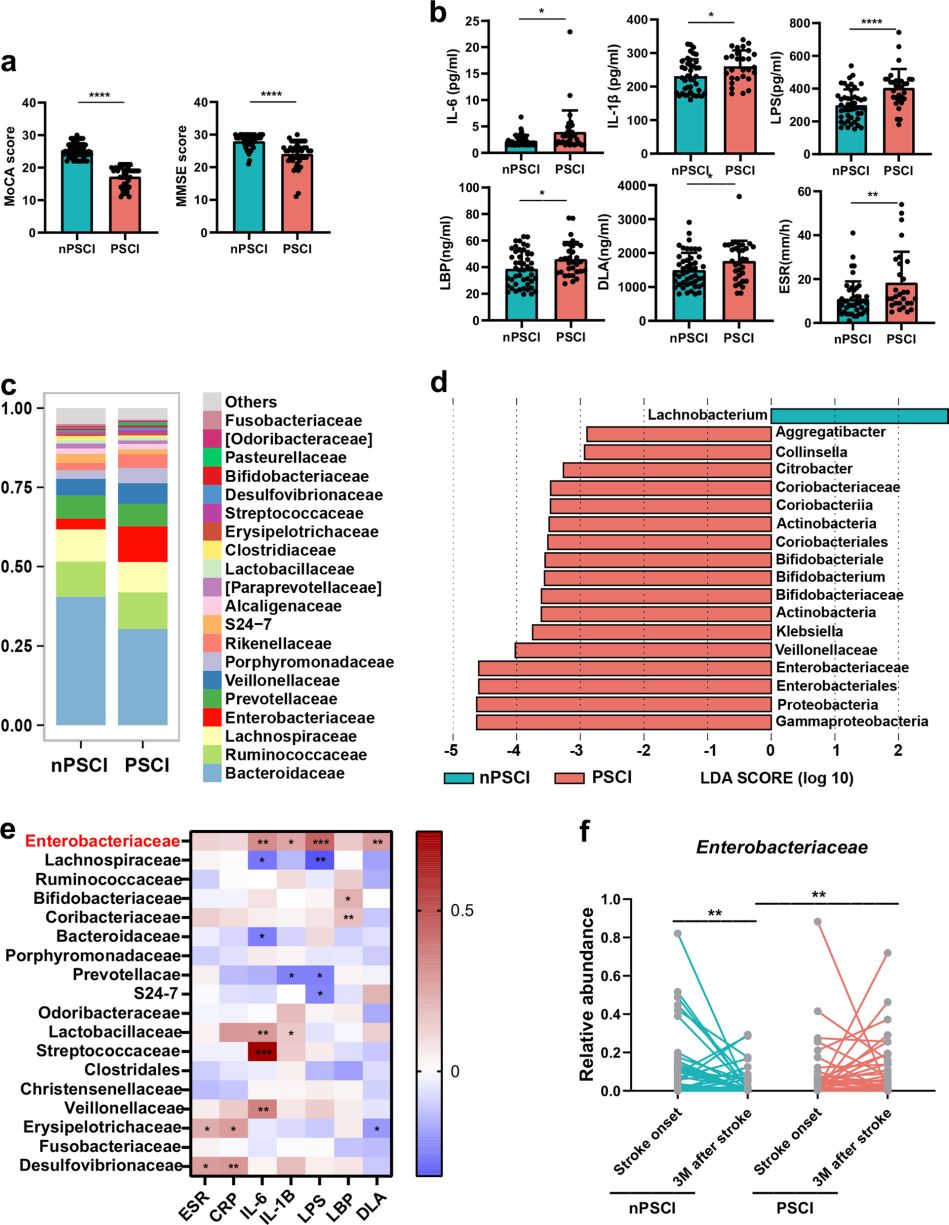
PSCI patients exhibit excessive peripheral inflammation and increased abundance of *Enterobacteriaceae*. **a** Mini-Mental State Examination (MMSE) and the Montreal Cognitive Assessment scale (MoCA) score between poststroke cognitive impairment (PSCI) patients (*n* = 34) and non-PSCI patients (*n* = 49) 3 months after stroke. Lower scores indicate severer cognitive impairment. **b** Levels of interleukin-6 (IL-6), IL-1β, lipopolysaccharide (LPS), LPS-binding protein (LBP), d lactate (DLA) and erythrocyte sedimentation rate (ESR) in peripheral blood. **c** Average relative abundances of prevalent microbiota at the family levels in the two groups. **d** Linear discriminant analysis effect size (LEfSe) shows bacterial taxa with significantly different abundances between the two groups. **e** Heatmap shows the association between gut microbiota and markers inflammation by Spearman’s rank correlation. **f** Changes in the relative abundance of *Enterobacteriaceae* from stroke onset to 3 months after stroke. Data are expressed as mean ± SEM. Student’s *t* test comparing MoCA, MMSE, inflammatory factors and abundances of *Enterobacteriaceae* at the same timepoint between PSCI and nPSCI patients; Wilcoxon matched-pairs signed rank test comparing abundances of *Enterobacteriaceae* between two timepoints of PSCI or nPSCI patients, *P < .05, **P < .01, ***P < .001, ****P < .0001

In the present study, 83 patients were recruited; 34 of the subjects were PSCI patients (assessed using the MoCA score at 3 months after stroke onset) and 49 of the subjects were nPSCI patients. The demographic data of patients are listed in Additional file 1: Table S1, the PSCI patients were older, had higher proportion of female and smoking; we found no difference in drinking, body mass index, NIHSS score, Barthel index, and history of hypertension, hyperlipidemia, diabetes and stroke, and stroke cause, infarct location and diet types (Additional file 1: Table S1). The PSCI patients had significantly lower MoCA (17.25 ± 3.17 versus 25.44 ± 2.30) and MMSE (24.08 ± 4.03 versus 27.87 ± 1.99) scores than nPSCI patients (Fig. 1a). The PSCI patients had higher levels of peripheral inflammatory factors, such as IL-6, IL-1β, LPS, LBP and DLA, and a higher ESR than the nPSCI patients (Fig. 1b). We analyzed gut microbiota composition at the family level and found a higher abundance of *Enterobacteriaceae* in PSCI patients (Fig. 1c). This finding was further substantiated by the LEfSe analysis, which identified several taxa that show significant differences in abundance between the two groups (Fig. 1d). Then, we conducted a correlation analysis and discovered that the abundance of *Enterobacteriaceae* was positively associated with the levels of several inflammatory factors (Fig. 1e). We analyzed the abundance of *Enterobacteriaceae* at two timepoints, i.e., stroke onset and 3 months after stroke, and found that *Enterobacteriaceae* abundance tended to decline over time in nPSCI patients but tended to increase over time in PSCI patients (Fig. 1f). In addition, the abundance of *Enterobacteriaceae* did not differ between the two groups at stroke onset (Additional file 2: Fig. S1a), and PSCI patients had a significantly lower abundance of butyrate-producing bacteria and lower levels of butyrate than nPSCI patients at stroke onset (Additional file 2: Fig. S1b–d). Moreover, multivariate logistic regression analyses revealed that the abundance of *Enterobacteriaceae* at 3 m after stroke (OR, 1.476; 95% CI 1.089 to 1.690; *P* = 0.006) and the increase of its abundance is an independent risk factor of PSCI (OR, 1.313; 95% CI 1.069 to 1.612; *P* = 0.009) after adjusting for sex, age, history of diabetes, hypertension, hyperlipidemia, stroke, smoke and drink, NIHSS score, Barthel index, body mass index, stroke cause, infarct location and diet types (Additional file 1: Table S2).

### Stroke mice receiving FMT from PSCI patients present higher *Enterobacteriaceae* abundance and lower fecal butyrate level than mice receiving FMT from nPSCI patients

**Fig. 2:**
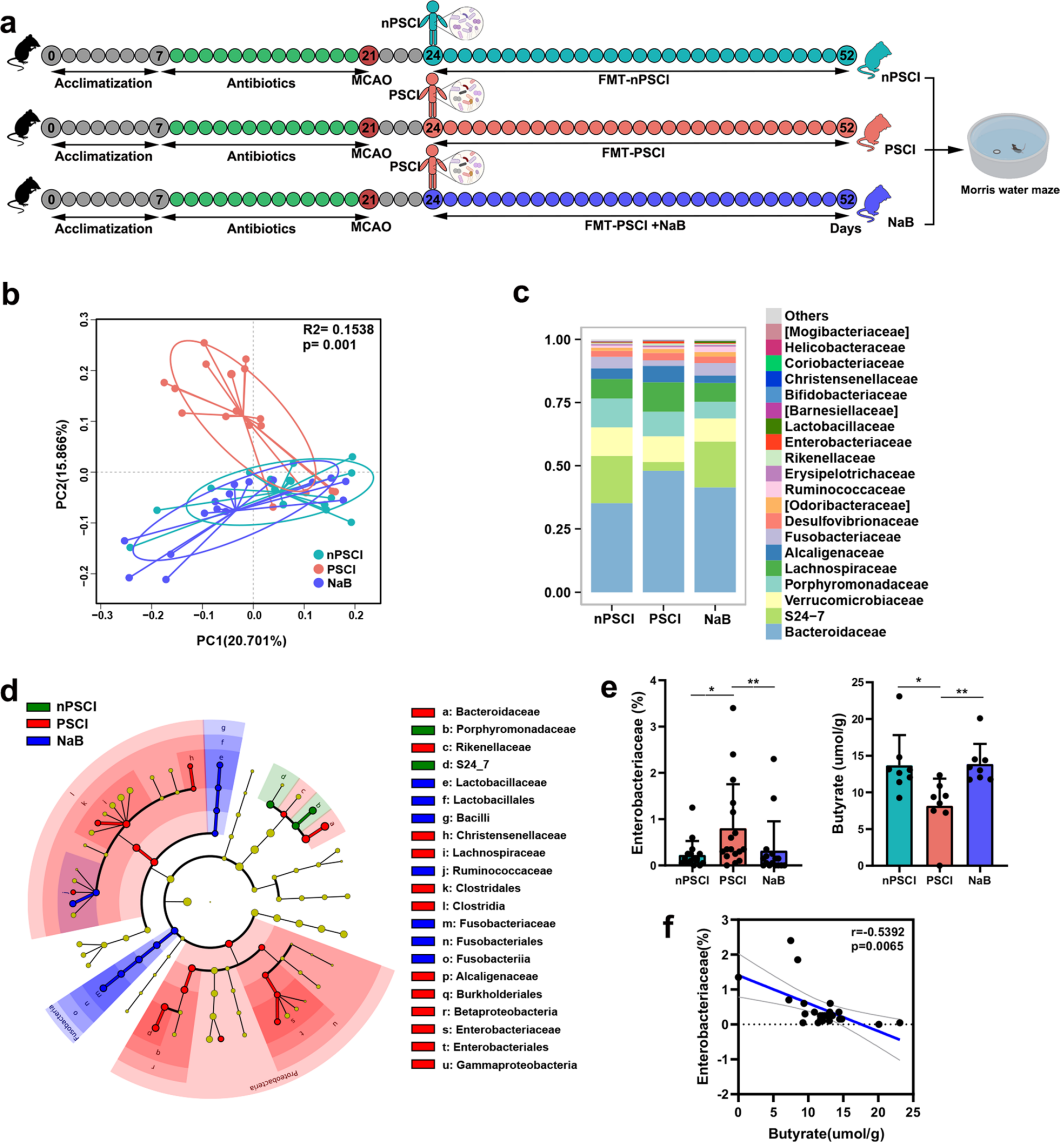
PSCI mice receiving present higher *Enterobacteriaceae* abundance and lower fecal butyrate level than nPSCI mice. **a** Experimental design. After acclimatization for 1 week, mice received antibiotics in the drinking water for 2 weeks, followed by middle cerebral artery occlusion (MCAO). 3 days after MCAO, gut microbiota from PSCI patients or non-PSCI patients was transferred to stroke mice by FMT. Sodium butyrate (NaB) was provided via drinking water. **b** Principal-coordinate analysis (PCoA) plot of unweighted UniFrac distances between the three groups. **c** Average relative abundances of prevalent microbiota at the family levels in the three groups. **d** LEfSe shows bacterial taxa with significantly different abundances between the three groups. **e** Relative abundances of *Enterobacteriaceae* and fecal butyrate levels in the three groups. **f** Correlations between *Enterobacteriaceae* and butyrate by Spearman’s rank correlation. Data are expressed as mean ± SEM, *n* = 15–17 mice per group, Nonparametric Kruskal–Wallis test comparing the abundance of *Enterobacteriaceae* and fecal butyrate level. *P < .05, **P < .01

To validate the role of the gut microbiota in the pathogenesis of PSCI, we transferred gut microbiota from PSCI patients into stroke mice. After acclimatization for 1 week, mice received antibiotics via drinking water for 2 weeks. The α- and β-diversity of the gut microbiota were drastically altered after antibiotic treatment (Additional file 2: Fig S2a, b). Then, the mice were subjected to experimental stroke. The mNSS was assessed 3 days after stroke, and then FMT from PSCI patients (PSCI mice) or nPSCI patients (nPSCI mice) was performed daily for 1 month (Fig. 2a). The PCoA plot of unweighted UniFrac distances revealed that the gut microbiota profile of PSCI mice was clearly separate from that of nPSCI mice (Fig. 2b). The average relative abundances of prevalent microbiota at the family level were used to determine the gut microbiota compositions of the mice (Fig. 2c), and LEfSe showed that the abundance of *Enterobacteriaceae* was significantly higher in PSCI mice than in nPSCI mice (Fig. 2d). In addition, the PSCI mice had a lower level of fecal butyrate than nPSCI mice (Fig. 2e). Correlation analysis revealed a negative association between the abundance of *Enterobacteriaceae* and the level of fecal butyrate (Fig. 2f). NaB treatment modulated the PSCI-associated gut microbiota towards the nPSCI-associated gut microbiota (Fig. 2b–d), decreased the abundance of *Enterobacteriaceae* and increased butyrate level (Fig. 2e).

### Stroke mice receiving FMT from PSCI patients display cognitive decline

**Fig. 3:**
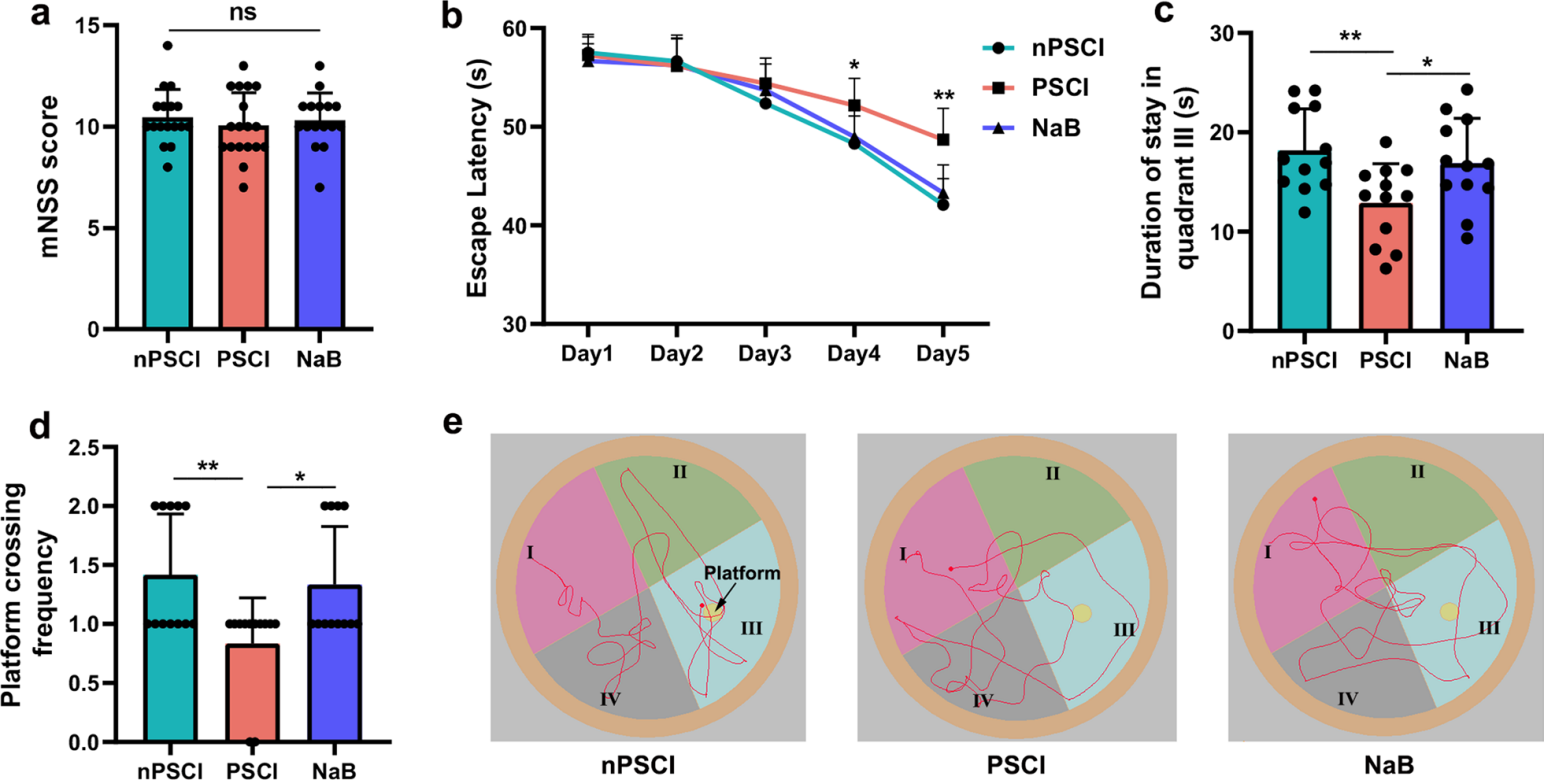
Stroke mice receiving FMT from PSCI patients display cognitive decline. **a** Modified Neurological Severity Score (mNSS) 3 days after stroke. **b** Escape latency of the three groups from days 1 to 5 by Morris water maze. **c** Duration of stay in quadrant III of the three groups. **d** Platform crossing frequency of the three groups. **e** Representative images of motion traces of the three groups. Data are expressed as mean ± SEM, *n* = 10–12 mice per group, one-way ANOVA comparing mNSS, duration of stay and platform crossing frequency. Escape latency was compared by repeated-measure ANOVA, ns, not significant, *P < .05, **P < .01

Three days after experimental stroke, the mNSS was assessed to evaluate stroke injury. mNSS were not significantly different between the two groups (Fig. 3a). After FMT, the Morris water maze was performed to assess the learning and memory of the mice. Mice in each group showed a downward trend in escape latency from day 1 to day 5, with PSCI mice showing worse spatial learning performance than nPSCI mice on days 4 and 5 (Fig. 3b). In addition, PSCI mice spent significantly less time in quadrant III and crossed the platform location fewer times than nPSCI mice (Fig. 3c–e). NaB treatment was efficient to improved cognitive decline of PSCI mice (Fig. 3b–e).

### PSCI-associated gut microbiota promotes hippocampal apoptosis and thalamic Aβ deposition

**Fig. 4:**
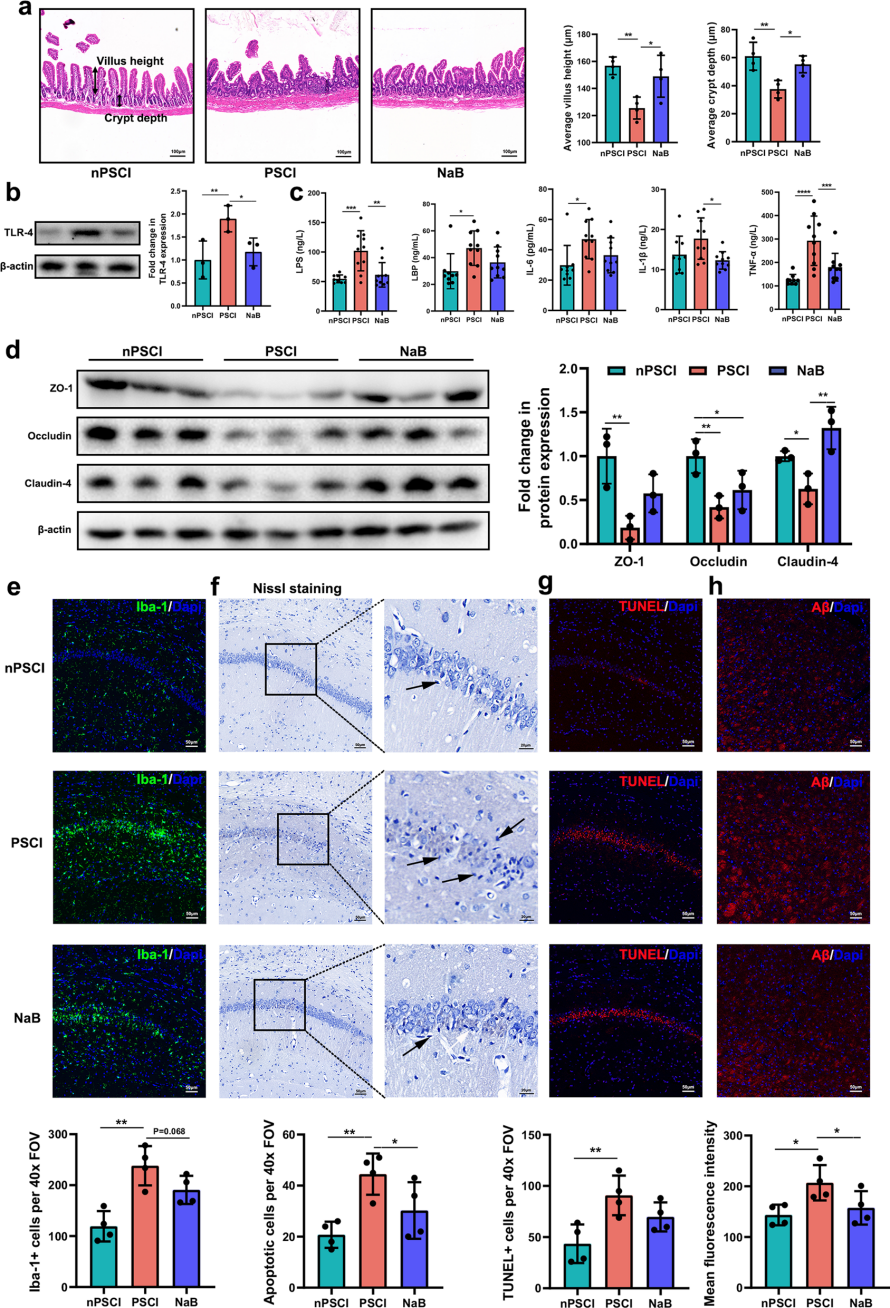
PSCI-associated gut microbiota promotes hippocampal apoptosis and thalamic Aβ deposition. **a** Morphology of the ileum was assessed using H&E staining (scale bar = 100 μm) and the average villus height and crypt depth was analyzed. **b** Expression of intestinal TLR-4 protein in the three groups. **c** Levels of LPS, LBP, IL-6, IL-1β and TNF-α in the peripheral blood. **d** Expression of cerebral tight junction proteins ZO-1, Occludin and Claudin-4 in the three groups. **e** Double immunostaining for Iba-1 (microglial marker) and **f** Nissl staining, and **g** TUNEL staining were performed in the hippocampal CA1 region to detect apoptotic neurons, and **h** Aβ staining was performed in the thalamus (scale bar = 50 μm). The cells were counted per 40× field of view (FOV). Data are expressed as mean ± SEM, *n* = 10–12 mice per group, one-way ANOVA, *P < .05, **P < .01, ***P < .001, ****P < .0001

To investigate the underlying mechanism of cognitive decline induced by PSCI-associated gut microbiota, we analyzed the morphology of the intestine. We found that PSCI mice exhibited epithelial disruption with a shorter villus height and crypt depth (Fig. 4a). We also found that the intestinal expression of TLR4, a receptor that recognizes LPS, was significantly higher in PSCI mice (Fig. 4b). Consistently, peripheral levels of LPS and LBP, along with the inflammatory factors IL-6, IL-1β and TNF-α were significantly higher in PSCI mice than in nPSCI mice (Fig. 4c). Furthermore, we found that the expression of tight-junction proteins associated with the blood–brain barrier (BBB), e.g., ZO-1, Occludin and Claudin-4 was significantly decreased in PSCI mice (Fig. 4d). Using immunofluorescence staining, we observed prominent microglial activation in the hippocampi of PSCI mice (Fig. 4e). Nissl staining showed significant neuronal loss and neuronal karyopyknosis and shrinkage of cell bodies in the hippocampal CA1 region in PSCI mice (Fig. 4f). TUNEL staining showed prominent neuronal apoptosis in the hippocampal CA1 region in PSCI mice (Fig. 4g). Notably, stroke mice did not exhibit significant Aβ deposition in the hippocampus (Additional file 2: Fig. S3a) but showed Aβ accumulation in the thalamus (Fig. 4h). Supplementation with NaB rescued these pathological changes in PSCI mice (Fig. 4a–h).

### Intraperitoneal injection of LPS after stroke causes similar pathology to those seen in mice receiving PSCI-associated gut microbiota

**Fig. 5:**
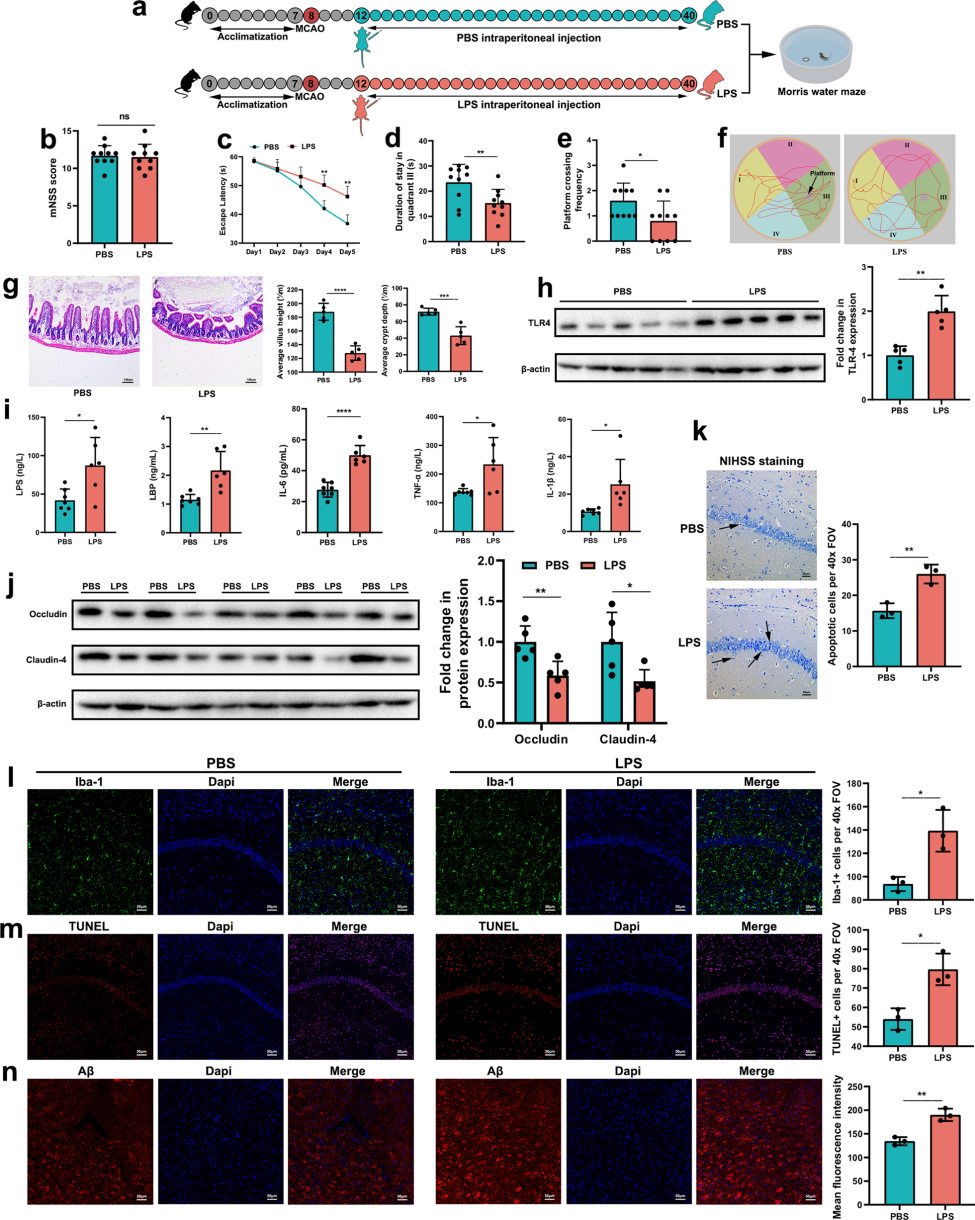
Intraperitoneal injection of LPS after stroke causes similar pathology to those seen in mice receiving PSCI-associated gut microbiota. **a** Experimental design. After acclimatization for 1 week, mice were subjected to MCAO. Three days after MCAO, mNSS score was assessed, followed by intraperitoneal injection of PBS or LPS daily. After 1 month, Morris water maze was performed after which mice were sacrificed. **b** Results of the mNSS score and **c**–**f** Morris water maze between the two groups. **g** Morphology of the ileum was assessed using H&E staining (scale bar = 100 μm) and the average villus height and crypt depth was analyzed. **h** Expression of intestinal TLR-4 protein in the two groups. **i** Levels of LPS, LBP, IL-6, IL-1β and TNF-α in the peripheral blood. **j** Expression of cerebral tight junction proteins Occludin and Claudin-4 in the two groups. **k** Nissl staining in the hippocampal CA1 region of the two group. **l** Double immunostaining for Iba-1 and **m** TUNEL staining in the hippocampal CA1 region, and **n** Aβ staining in the thalamus (scale bar = 50 μm). The cells were counted per 40× FOV. Data are expressed as mean ± SEM, *n* = 9–10 mice per group, Student’s *t* test, escape latency was compared by repeated-measure ANOVA, ns, not significant, *P < .05, **P < .01, ***P < .001, ****P < .0001

To further demonstrate the role of LPS in the pathogenesis of PSCI, stroke mice were intraperitoneally injected with LPS (LPS mice) or PBS (PBS mice) (Fig. 5a). mNSSs were not different between the two groups 3 days after stroke (Fig. 5b). However, LPS mice showed worse spatial learning performance than PBS mice on days 4 and 5 of the trials (Fig. 5c). Moreover, LPS mice spent less time in quadrant III and crossed the platform fewer times than PBS mice (Fig. 5d–f). LPS mice also exhibited intestinal morphology damage (Fig. 5g) and higher intestinal expression of TLR4 (Fig. 5h) than PBS mice. In addition, the peripheral levels of the inflammatory factors LPS, LBP, IL-6, TNF-α and IL-1β were also higher in LPS mice than in PBS mice (Fig. 5i). As expected, LPS mice exhibited lower expression of tight-junction proteins associated with the BBB and more neuronal loss than PBS mice (Fig. 5j, k). Furthermore, LPS mice showed more microglial activation, and neuronal apoptosis in the CA1 region of the hippocampus and more Aβ deposition in the thalamus (Fig. 5l, m). Taken together, these data suggest that intraperitoneal injection of LPS after stroke causes similar pathology to those seen in mice receiving PSCI-associated gut microbiota.

## Discussion

PSCI is highly prevalent among stroke patients, and a systemic inflammatory state has been observed in PSCI patients [2, 4–6]. However, how systemic inflammation develops in PSCI patients remains to be elucidated. In the current study, we show for the first time that the gut microbiota-derived metabolites, i.e., LPS and butyrate, mediate the crosstalk between the gut and the brain in the context of PSCI.

We observed higher levels of inflammatory factors in PSCI patients than in nPSCI patients and identified several taxa that showed significant differences in abundance between the two groups, i.e., *Gammaproteobacteria*, *Proteobacteria*, *Enterobacteriaceae*, et al., which is consistent with previous studies [19–21]. We further performed a correlation analysis and found that *Enterobacteriaceae* abundance was the strongest indicator of inflammation, while the abundance of *Lachnospiraceae* (a butyrate-producing bacteria) showed a negative association with inflammation. Interestingly, we found that the abundance of *Enterobacteriaceae* tended to increase over time in PSCI patients, whereas it tended to decrease over time in nPSCI patients. Considering that the abundance of *Enterobacteriaceae* and NIHSS scores did not differ between the two groups but the abundances of butyrate-producing bacteria and fecal butyrate were significantly reduced in the PSCI group at stroke onset, we hypothesized that the increase in peripheral LPS levels and subsequent inflammation might be due to disruption of the gut barrier, because there is compelling evidence suggesting an important role for butyrate in maintaining intestinal barrier function and ameliorating intestinal inflammation [30–34]. Therefore, we administered NaB to PSCI mice via drinking water to protect the intestinal barrier. In our previous study, we found that NaB supplementation can modulate the gut microbiota and decrease the LPS level in diabetic mice with stroke [28]. Surprisingly, in the present study, we found that NaB treatment altered the PSCI-associated gut microbiota so that it was similar to the nPSCI-associated gut microbiota according to the PCoA plot. NaB decreased the abundance of *Enterobacteriaceae* and increased fecal butyrate levels in PSCI mice. Furthermore, fecal butyrate concentrations showed a negative correlation with *Enterobacteriaceae* abundance. Therefore, NaB may represent a potential therapeutic agent for decreasing the abundance of *Enterobacteriaceae* in PSCI patients.

Stroke mice receiving PSCI-associated gut microbiota exhibited intestinal disruption and higher protein expression of TLR4, a receptor that recognizes LPS. LPS causes inflammatory activation mainly by binding TLR4 and MD2 on the surface of the cell, which activates nuclear factor κB (NFκB), thereby promoting the transcription of many inflammatory factors including IL-6, IL-1β and TNF-α [35, 36]. LBP is a plasma protein that cooperates with CD14 to facilitate the transfer of LPS to TLR4 [37]. Although LPS is a major producer of members of *Enterobacteriaceae*, which includes the genera *Escherichia* and *Klebsiella*, it is not the only bacteria that produces LPS. Different species of gram-negative bacteria possess different LPS structures, i.e., the O-antigen and lipid A [38]. Therefore, we intraperitoneally injected stroke mice with LPS derived from *Escherichia coli*, which is thought to be the most inflammatory form of LPS [39]. Intraperitoneal injection of LPS caused an equivalent level of peripheral LPS and similar pathology as transfer of gut microbiota from PSCI patients. In a recent study, researchers found that compared with mice receiving FMT from saline-treated mice, mice receiving FMT from recombinant human atrial natriuretic peptide (rhANP)-treated mice exhibited attenuated systemic and cerebral inflammation, and improved cognitive function after intraperitoneal LPS injection [40]. The authors proposed that the gut microbiota might play an essential role in the rhANP-mediated amelioration of cognitive impairment caused by LPS, while acknowledging that they did not examine the gut microbiota profile of the mice after rhANP treatment. Based on the findings of our study, we speculate that the *Enterobacteriaceae* family or butyrate-producing bacteria may be involved in rhANP-mediated changes in the gut microbiota. However, further studies are needed to address this issue.

**Fig. 6:**
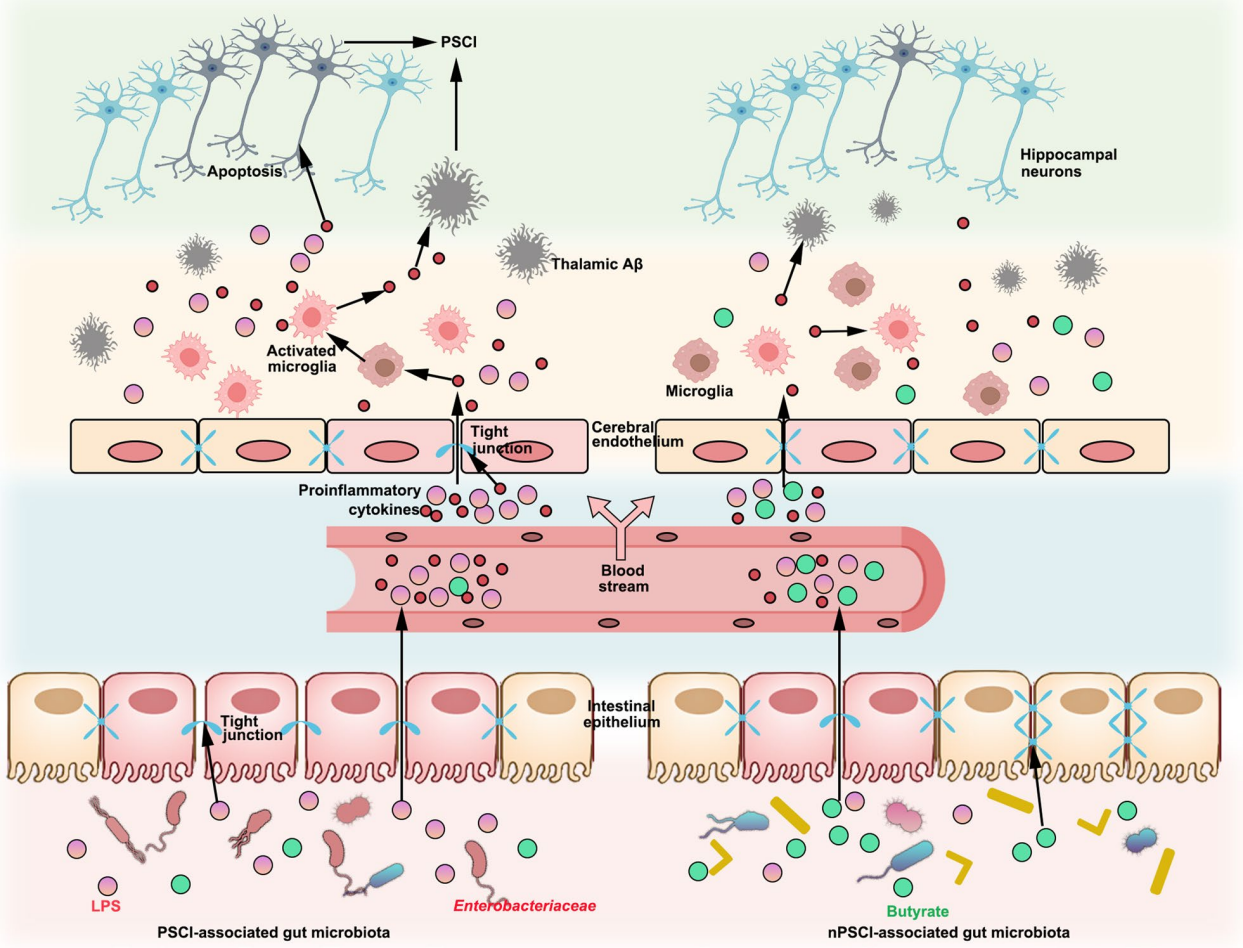
PSCI-associated gut microbiota promotes hippocampal apoptosis and thalamic Aβ deposition. The gut microbiota of PSCI patients is hallmarked by an increased abundance of *Enterobacteriaceae* and a decreased level of butyrate, which result in a disrupted gut barrier. The LPS constantly travels through the leaky gut into circulation, causing a chronic peripheral inflammation. The continuous inflammation destroys the integrity of BBB, leading to constant infiltration of peripheral LPS and inflammatory cytokines, which promote the neuronal apoptosis in the CA1 region of hippocampus, a brain region that is critical for the cognition. In addition, the low-grade chronic inflammation in the brain promotes the deposition of Aβ plaque in the thalamus, a toxic chemical that is associated with cognitive impairment

Given that the hippocampus plays a critical role in processing of spatial information and memory formation, pathological changes in the hippocampus, including neuroinflammation caused by ischemic stroke, have drawn increasing attention from researchers [41]. Stroke and Alzheimer’s disease (AD) share many common risk factors, such as hypertension, diabetes, and hyperlipidemia [42]. In addition, PSCI and AD have been implicated to have some common molecular pathologies [43]. Cerebral Aβ deposition is one of the hallmarks of AD and is frequently accompanied by cerebrovascular pathology in AD patients [44]. Most rodent studies have revealed that Aβ is deposited in the thalamus after stroke, while some studies have found Aβ is deposited in the hippocampus [43]. In our study, we did not observe apparent Aβ deposition in the hippocampus, but we noticed excessive Aβ accumulation in the thalami of the mice. This may have been, because the deposition of Aβ after stroke can change in distribution over time [45], resulting from plaque-forming or clearing enzyme [46, 47]. Aβ is a neurotoxic protein that is positively associated with PSCI [48]. Evidence suggests that Aβ oligomers contribute to neuronal loss after stroke via a feedforward neurodegeneration loop [49]. Moreover, Aβ oligomers released from ischemic cells are able to trigger microglial activation to induce a proinflammatory state, leading to neuronal death [50]. Importantly, the hippocampus is highly interconnected with thalamic nuclei, e.g., the anterior and mediodorsal nuclei, which are known to play an important role in cognitive function [51]. Based on our findings, the PSCI-associated gut microbiota may contribute to cognitive impairment through hippocampal apoptosis and thalamic Aβ deposition (Fig. 6).

We acknowledge several limitations to this study. First, the sample size of the recruited patients in this study is relatively small. Further studies with more samples are needed to confirm the results. Second, although we believe that gut-derived LPS plays a critical role in the pathogenesis of PSCI, it will be necessary to use TLR4-knockout mice to substantiate the underlying mechanism. Third, we could not exclude the effects of other pathogens on PSCI, since we utilized the whole fecal contents and we could not exclude the effects of Na^+^ that derived from NaB on the gut microbiota. Fourth, we only used filament MCAO model in this study, different stroke model should be used to further verify these findings.

## Conclusions

In summary, our study reveals a proinflammatory state in PSCI patients and shows that gut microbiota-derived LPS underlies inflammation, with the *Enterobacteriaceae* family possibly being the major contributor. Through FMT, we found that the PSCI-associated gut microbiota disrupts the intestinal barrier and elevates LPS and inflammation, which exacerbates BBB destruction, microglial activation, hippocampal apoptosis and thalamic Aβ deposition, leading to cognitive dysfunction in stroke mice. Supplementation with NaB ameliorates the above damage induced by PSCI-associated gut microbiota and is thus a potential therapeutic strategy for PSCI.

## Supplementary Information

**Additional file 1: Table S1.** Characteristics of the patients. **Table S2.** Multivariate logistic regression analyses.

**Additional file 2: Figure S1.** Gut microbiota profile of the PSCI and non-PSCI patients at stroke onset. **Figure S2.** α- and β-diversity of the gut microbiota before and after antibiotics treatment. **Figure S3.** Aβ deposition in the hippocampus of mice.

## Data Availability

The data sets used and/or analyzed during the current study are available from the corresponding author on reasonable request.

## Abbreviations

PSCI: Poststroke cognitive impairment
MoCA: Montreal Cognitive Assessment
FMT: Fecal microbiota transplantation
BBB: Blood–brain barrier
Aβ: β-Amyloid
LPS: Lipopolysaccharide
TLR4: Toll-like receptor-4
LBP: LPS-binding protein
NaB: Sodium butyrate
CRP: C reactive protein
IL: Interleukin
NIHSS: National Institutes of Health Stroke Scale
MMSE: Mini-Mental State Examination
MCAO: Middle cerebral artery occlusion
mNSS: Modified neurological severity score
PD: Phylogenetic diversity
PCoA: Principal-coordinate analysis
LEfSe: Linear discriminant analysis effect size
LDA: Linear discriminant analysis
SCFA: Short-chain fatty acid
DLA: D lactate
ESR: Erythrocyte sedimentation rate
ELISA: Enzyme-linked immunosorbent assay
PFA: Paraformaldehyde
DAPI: 4ʹ,6-Diamidino-2-phenylindole
PMSF: Protease inhibitor phenylmethylsulfonyl fluoride
NFκB: Nuclear factor κB
rhANP: Recombinant human atrial natriuretic peptide
AD: Alzheimer’s disease
