# Supplementary material for: Gut microbiota is causally associated with poststroke cognitive impairment through lipopolysaccharide and butyrate
Source: J Neuroinflammation. 2022 Apr 4;19:76. doi: 10.1186/s12974-022-02435-9 (PMC8981610; doi:10.1186/s12974-022-02435-9)
Supplement: Supplementary file 1 — Additional file 1: Table S1. Characteristics of the patients. Table S2. Multivariate logistic regression analyses. [file 12974_2022_2435_MOESM1_ESM.docx]

Table S1 Characteristics of the patients.

| **Characteristics*** | 3 months after onset, MoCA ＜22 | | |
| --- | --- | --- | --- |
|  | PSCI | nPSCI | P |
| **No. of subjects** | 34 | 49 |  |
| **Demographics** |  |  |  |
| Age, y | 61.5 (13) | 54.0 (11) | 0.000 |
| Female (%) | 10 (35.3) | 5 (10.2) | 0.025 |
| Cigarette smoking (%) | 15 (44.1) | 34 (69.4) | 0.021 |
| Alcohol drinking (%) | 11 (32.4) | 19 (38.8) | 0.549 |
| **Clinical features** |  |  |  |
| Body mass index, kg/m^2^ | 23.7 (3.6) | 23.9 (3.8) | 0.386 |
| Baseline NIHSS score | 2 (3) | 2 (2) | 0.509 |
| Baseline Barthel index | 75 (40) | 85 (35) | 0.657 |
| **Medical histories** |  |  |  |
| History of hypertension | 24 (70.6) | 30 (61.2) | 0.379 |
| History of hyperlipidemia | 10 (29.4) | 17 (34.7) | 0.613 |
| History of diabetes | 12 (35.3) | 15 (30.6) | 0.654 |
| History of stroke | 6 (17.6) | 2 (4.1) | 0.059 |
| **Stroke causes** |  |  |  |
| Large artery atherosclerosis | 14 (41.2) | 29 (59.2) | 0.974 |
| **Cortex infarction** | 11 (32.4) | 17 (34.7) | 0.824 |
| **Diet types** |  |  | 0.239 |
| meat | 14 (41.2) | 19 (38.8) |  |
| Vegetarian | 10 (29.4) | 8 (16.3) |  |
| mixed | 10 (29.4) | 22 (44.9) |  |

No. (%) or median value (IQR)

Table S2 Multivariate logistic regression analyses.

|  | 3 months after onset | |
| --- | --- | --- |
|  | Adjusted OR (95% CI) | P value |
| **Enterobacteriaceae*  Stroke onset | 0.978 (0.804-1.189) | 0.821 |
| **Enterobacteriaceae*  3m after stroke | 1.357 (1.089-1.690) | 0.006 |
| Δlog *Enterobacteriaceae* | 1.313 (1.069-1.612) | 0.009 |

Adjusted for sex; age; history of diabetes, hypertension, hyperlipidemia, stroke, smoke and drink; NIHSS score; Barthel index; body mass index; stroke cause; infarct location; diet types.

* log2-transformed
