# Supplementary material for: Gut microbiota is causally associated with poststroke cognitive impairment through lipopolysaccharide and butyrate
Source: J Neuroinflammation. 2022 Apr 4;19:76. doi: 10.1186/s12974-022-02435-9 (PMC8981610; doi:10.1186/s12974-022-02435-9)
Supplement: Supplementary file 2 — Additional file 2: Figure S1. Gut microbiota profile of the PSCI and non-PSCI patients at stroke onset. Figure S2. α- and β-diversity of the gut microbiota before and after antibiotics treatment. Figure S3. Aβ deposition in the hippocampus of mice. [file 12974_2022_2435_MOESM2_ESM.docx]

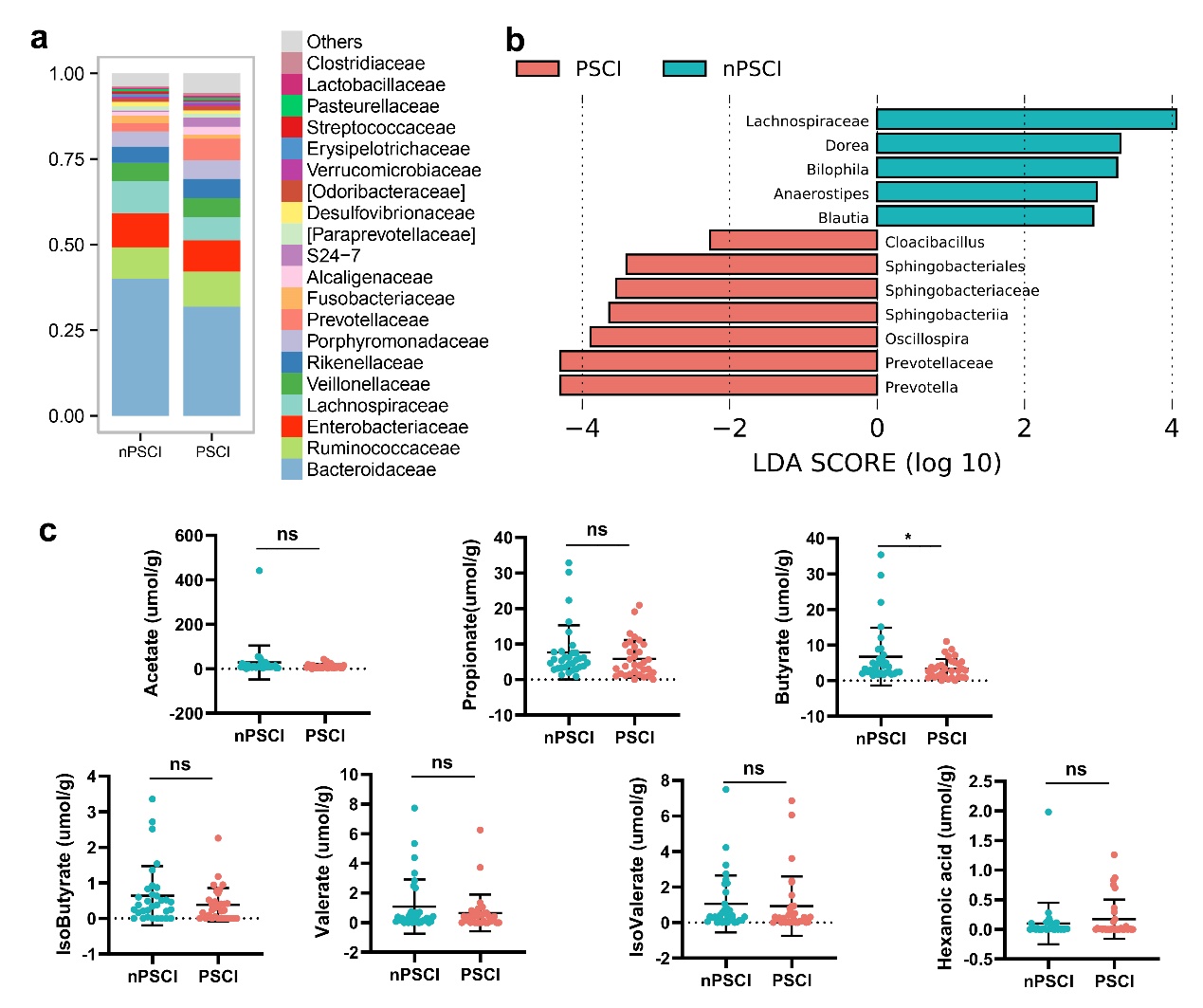


**Fig. S1** The gut microbiota profile of the PSCI and non-PSCI patients at stroke onset. (a) Average relative abundances of prevalent microbiota at the family levels in the two groups. (b) The linear discriminant analysis effect size (LEfSe) shows bacterial taxa with significantly different abundances between the two groups. (c) Levels of fecal short-chain fatty acids (SCFAs) in the two groups. Data are expressed as mean ± SEM, nonparametric Kruskal-Wallis test. *p < .05.


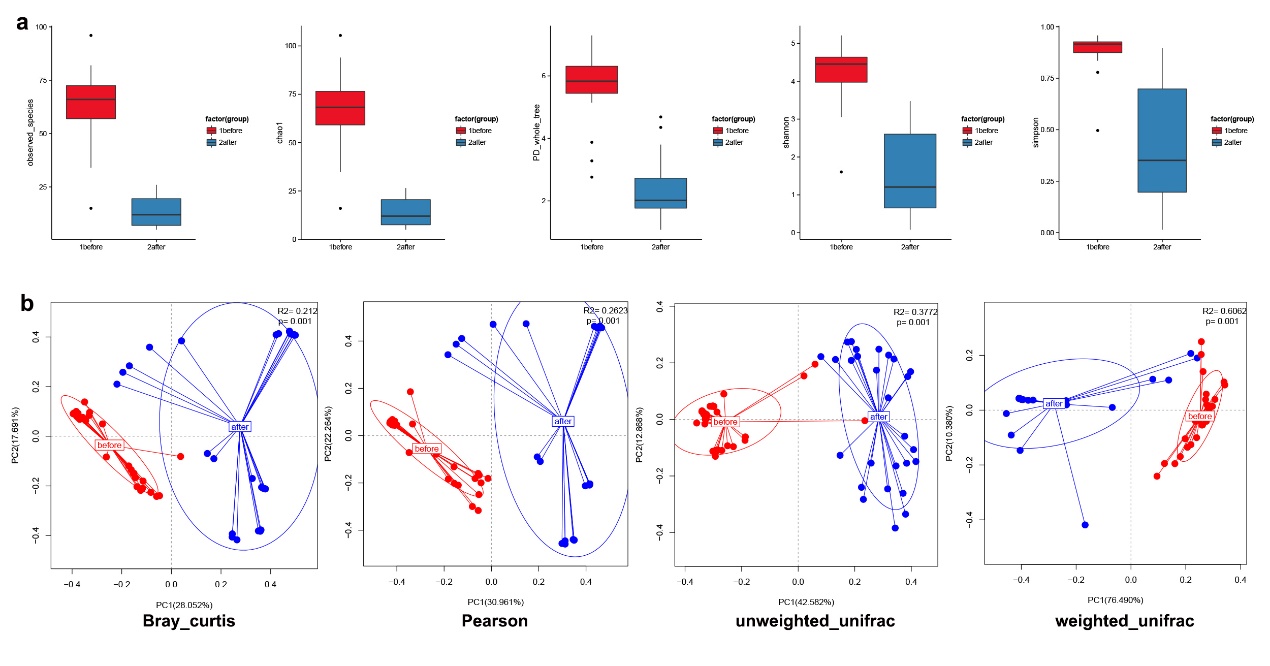


**Fig. S2** The α- and β-diversity of the gut microbiota before and after antibiotics treatment. (a) The α-diversity indices observed_species, chao1, PD_whole_tree, shannon and simpson index. (b) The β-diversity of the gut microbiota before and after antibiotics treatment.


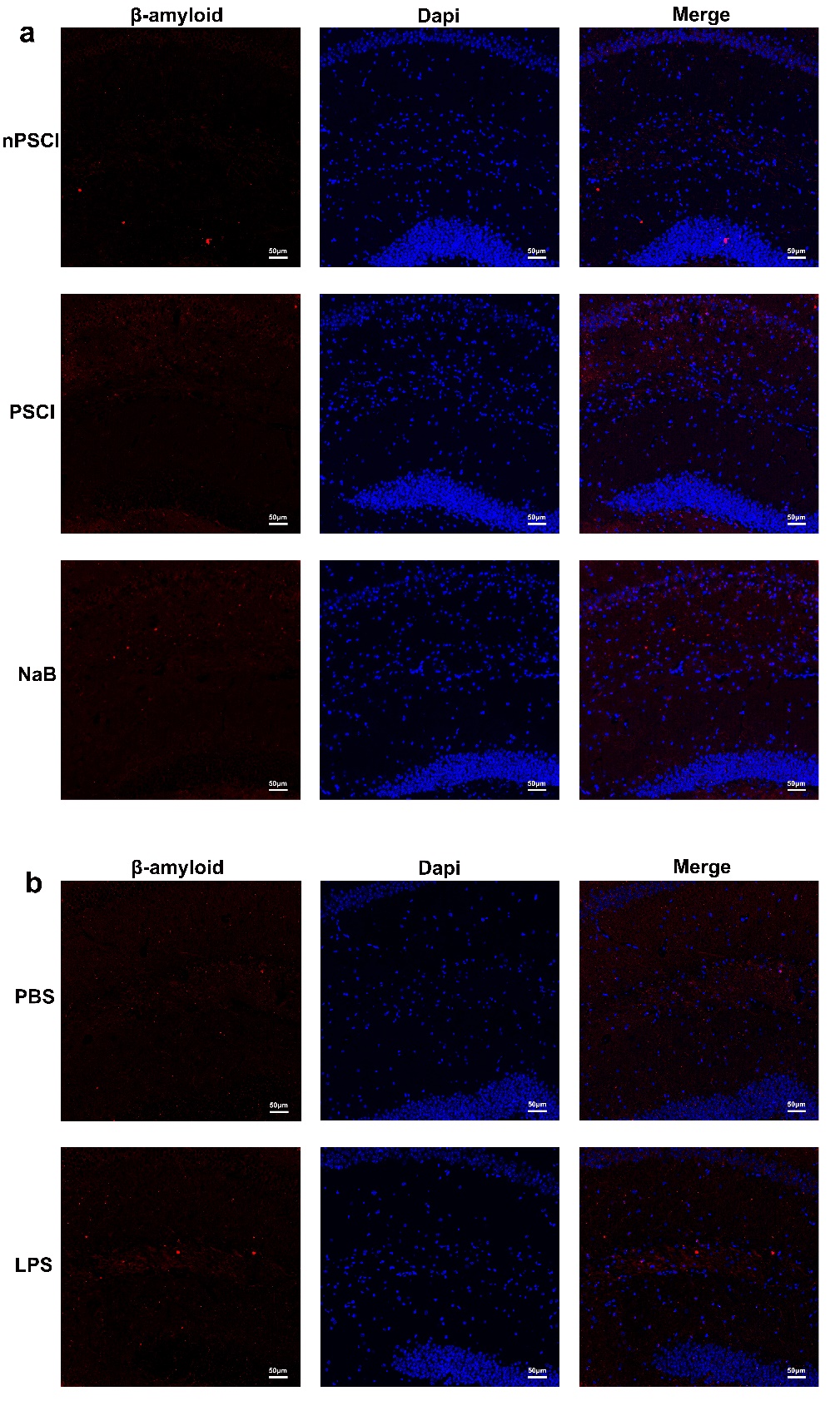


**Fig. S3** The Aβ deposition in the hippocampus of mice. (a) The Aβ deposition in the hippocampus of mice receiving FMT from PSCI (with or without NaB treatment) or nPSCI patients. (b) The Aβ deposition in the hippocampus of mice with PBS or LPS treatment.
